# Supplementary material for: An O-GlcNAcylomic Approach Reveals ACLY as a Potential Target in Sepsis in the Young Rat
Source: Int J Mol Sci. 2021 Aug 26;22(17):9236. doi: 10.3390/ijms22179236 (PMC8430499; doi:10.3390/ijms22179236)
Supplement: Supplementary file 1 [file ijms-22-09236-s001.zip › ijms-1329913-Supplementary files-final/Supplementary files/Supplementary Material reviewing.pdf]

Supplementary material

An *O*-GlcNAcylomic approach reveals ACLY as a potential target in sepsis in the young rat

Manon Denis[1,2], Thomas Dupas[1], Antoine Persello[1,3], Justine Dontaine[4], Laurent Bultot[4], Charlotte Betus[1], Thomas Pelé[1], Justine Dhot[1,5], Angélique Erraud[1], Anaïs Maillard[1], Jérôme Montnach[1], Aurélia A. Leroux[1,6], Edith Bigot-Corbel[7], Didier Vertommen[8], Matthieu Rivière[9], Jacques Lebreton[9], Arnaud Tessier[9], Michel De Waard[1], Luc Bertrand[4,10], Bertrand Rozec[1], Benjamin Lauzier[1]

From the [1] Université de Nantes, CHU Nantes, CNRS, INSERM, l'institut du thorax, F-44000 Nantes, France ; [2] Pediatric Intensive Care Unit, CHU de Nantes, Nantes, France ; [3] InFlectis BioScience, Nantes, France ; [4] Université catholique de Louvain, Institut de Recherche Expérimentale et Clinique, Pôle of Cardiovascular Research, Brussels, Belgium ; [5] Sanofi R&D, 1 Avenue Pierre Brossolette, Chilly Mazarin, France ; [6] University Animal Hospital, Oniris Ecole Nationale Vétérinaire, Agroalimentaire et de l'Alimentation Nantes Atlantique, Nantes, France ; [7] Departement of Biochemistry, CHU de Nantes, Nantes, France ; [8] Université catholique de Louvain, de Duve Institute, Mass Spectrometry Platform, Brussels, Belgium ; [9] Université de Nantes, CNRS, Chimie et Interdisciplinarité: Synthèse, Analyse, Modélisation (CEISAM), UMR CNRS 6230, Faculté des Sciences et des Techniques, Nantes, France ; [10] WELBIO, Brussels, Belgium.

Corresponding author. Tel: +33 2 28 08 01 57; E-mail: benjamin.lauzier@univ-nantes.fr; address: 8 quai Moncousu, l'institut du thorax, 44007 Nantes, France.

Methods (supplemental)

2.2. Animal models and measures

Thirty minutes before shock induction, rats received intravenous buprenorphine (0.03 mg/kg). Endotoxemic shock was then induced in four-week-old male Wistar rats (Charles River, Saint Germain Nuelles, France) by intravenous injection of lipopolysaccharides (LPS, 20 mg/kg, LPS from *E. Coli* O111:B4, *Sigma*, France) and compared with control rats (*i.v.* injection of saline). One hour later, LPS rats received no treatment (LPS) or were resuscitated with subcutaneous injection of saline 10 mL/kg alone (LPS+R) or completed with NButGT (10 mg/kg) (**Figure S1**). Three hours after LPS injection, rats were investigated for *in vivo* measurements and hearts were then freeze clamped for tissue samples. In order to avoid circadian clock modification in protein *O*-GlcNAcylation, the shock induction was performed in the mornings between 8:30 and 10:15 a.m.

Blood gas parameters from arterial samples were analyzed using Gem Premier 3000 blood gas (Instrumentation Laboratory Werfen, Le Pré Saint Gervais, FRANCE). Lactate was also measured on venous blood samples using the Nova StatStrip® Lactate Point-of-Care (Nova Biomedical, Rungis, France). Serum creatinine was measured by an enzymatic method using creatininase and troponin Tc Hs measured using immunochemiluminescence on a Cobas 6000 Ce analyzer (Roche, Meylan, France).

The PRISM score was adapted to the animal model based on the PRISM (Pediatric RISk of Mortality) score used in pediatric intensive care units. It is a predictive factor of patient admission mortality. It is based on the analysis of a global health status (**Table S1A**) and different physiological and biological parameters (blood pressure, ventilatory parameters, leukocytes, lactates, blood sugar, pH etc.) (**Table S1B**).

A 36h survival study was conducted. Animals were randomized between the different groups (CTRL; LPS+R; NButGT; n = 16 per group). Scrupulous monitoring allowed for the best possible tracking of the animal. Each hour, health status was evaluated and euthanasia with lethal dose of pentobarbital (Dolethal®, Vetoquinol, Paris, France) was realized if limit points were reached or when the animals were judged to be in too impaired a general state of health (incapacity to move, *decubitus* position and difficulty to breathing) for three hours straight.

2.4. Western blot

The powder was solubilized on ice in lysis buffer (600µL/30mg powder) (**Table S2**). A second grinding was carried out using the TissueLyser (Qiagen) at 30Hz for 1.5 min. A centrifugation

at 10,000 g and 4°C was finally carried out for 5 min to precipitate the cellular debris. The supernatant, containing the proteins, was recovered to perform a protein assay. Western blotting experiments were performed on heart as previously described (1) (**Table E3**). Analysis was performed using Image Lab software (Bio-Rad).

2.5 Mass spectrometry and protein identification

2.5.1. Purification of *O*-GlcNAcylated proteins and LC-MS/MS

20 mg of heart powder were homogenized in 200 µL of RIPA lysis buffer (ThermoFisher Scientific Inc., Waltham, Massachusetts, United States, #89900) supplemented with a protease/phosphatase inhibitor cocktail (ThermoFisher Scientific Inc. #78430 and #78428) and 1 µM of *O*-GlcNAc cycling enzyme inhibitors (Sigma-Aldrich, Saint Louis, Missouri, United States, A7413 and A7229). Proteins from the lysate were precipitated using the chloroform/methanol precipitation method and then resuspended in 1% SDS and 20 mM HEPES buffer at pH 7.9. To completely dissolve the pellet, proteins were resuspended and heated 5-10 min at 90°C. *O*-GlcNAc groups from proteins were firstly stabilized and labelled with tetramethylrhodamine azide (TAMRA) by using the Click-iT® *O*-GlcNAc enzymatic labelling system kit (C33368) and followed by the Click-iT® protein analysis detection kit (C33370) from Invitrogen according to the manufacturer's instructions. Afterwards, SDS was quenched with NEFTD buffer (100 mM NaCl, 50 mM Tris-HCl pH 7.4, 5 mM EDTA, 6% NP-40) and lysate was precleared with washed protein G sepharose beads. Following centrifugation (500 g - 1 min), supernatant was incubated with pre-washed protein G sepharose beads (10 µL/500 µg of protein) coupled with anti-TAMRA antibody (15 µg/500 µg of protein, A6397, Invitrogen) for 1.5h at 4°C. After centrifugation (500 g - 1 min), the beads were washed with once NEFTD buffer and three times with NEFT buffer (NEFTD without NP-40). The beads were then boiled 5 min in Laemmli buffer (2 mM EDTA, 4% SDS, 20% Glycerol, 0.004% bromophenol blue, 50 mM DTT and 100 mM Tris pH 6.8). The proteins from above were separated on 1 mm thick criterion TGX, 4-15%, 26 wells (Bio-Rad, 567-1085) and stained with Colloidal Coomassie blue (Sigma-Aldrich). Gel bands were first in-gel digested with trypsin. Peptides separation was performed using a C18 reversed-phase analytical column (Thermo Scientific) on an Ultimate 3000-nLC RSLC system. The peptides were subjected to Nano-Spray-Ionization source followed by tandem mass spectrometry in a tribrid Fusion Lumos Orbitrap analyser coupled online to the nano-LC. Spectra were acquired by a data dependent scan routine with ion precursor detection in the Orbitrap and daughter ions in the Iontrap. The resulting MS/MS data were processed using Sequest HT search engine within Proteome

Discoverer 2.4 against a rat protein database obtained from Uniprot (29 953 entries). Trypsin was specified as cleavage enzyme allowing up to 2 missed cleavages, 4 modifications per peptide and up to 5 charges. Mass error was set to 10 ppm for precursor ions and 0.1 Da for-fragment ions. Oxidation on methionine, carbamidomethyl on cysteine were considered as variable modifications. False discovery rate (FDR) was assessed using Percolator and thresholds for protein, peptide and modification site were specified at 1%. The filtered Sequest HT output files for each peptide were grouped according to the protein from which they were derived and abundance was evaluated by label-free quantification within Proteome Discoverer from area under the curve of MS1 intensities. Finally, we identified 5593 putative *O*-GlcNAcylated proteins and for each protein.

2.5.2. LC-MS/MS analysis and protein-protein interactions

The 5593 proteins were analyzed using R software (version 3.12.0). Only 1327 proteins that have been identified with a molecular weight corresponding to the expected weight were selected. Only 48 proteins matched with selection criteria (at least 2 unique peptides, 15% of coverage and no missing values for each group). Differentially *O*-GlcNAcylated proteins were defined as those with log2 changes of at least 0.9-fold between 2 conditions. For proteins with differential *O*-GlcNAc levels, protein-protein interactions (PPIs) analysis has been performed using STRING database (v11) (2). Line thickness of PPIs indicates the strength of data support.

1. Merlet N, Piriou N, Rozec B, Grabherr A, Lauzier B, Trochu J-N, Gauthier C. Increased Beta2-Adrenoceptors in Doxorubicin-Induced Cardiomyopathy in Rat. *PLoS ONE* 2013;8:.
2. Szklarczyk D, Gable AL, Lyon D, Junge A, Wyder S, Huerta-Cepas J, Simonovic M, Doncheva NT, Morris JH, Bork P, Jensen LJ, Mering C von. STRING v11: protein–protein association networks with increased coverage, supporting functional discovery in genome-wide experimental datasets. *Nucleic Acids Res* 2019;47:D607–D613.

Figure (supplemental)

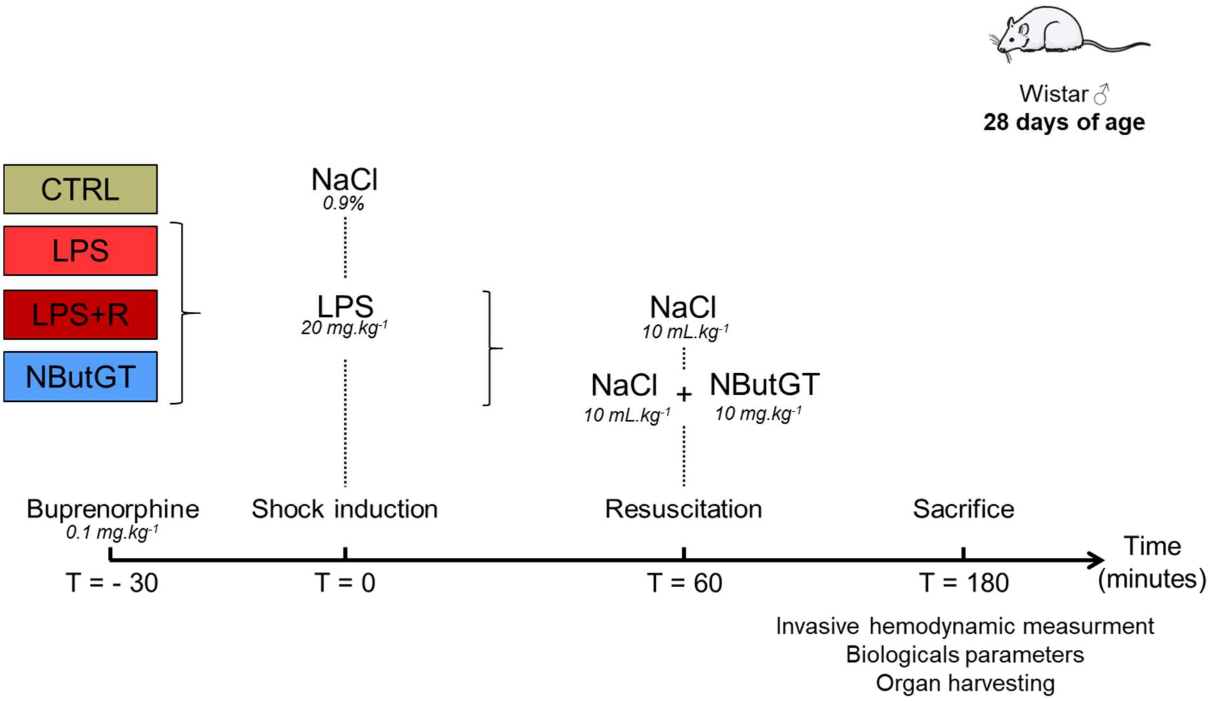

Figure S1. Protocol for endotoxemic shock and treatments

4 week-old animals were randomized in one of the four protocols (n = 20-22 per group), CTRL: control group, LPS: *i.v.* injection of LPS (*E. Coli* O111:B4, 20mg/kg), LPS+R: resuscitation with a subcutaneous administration of 10 $mL.kg^{-1}$ of NaCl solution 0.9% 1h after LPS injection, NButGT: addition of an OGA inhibitor to the fluid resuscitation (NButGT: 10 $mg.kg^{-1}$).

Tables (supplemental)

Table S1. Adapted PRISM score

A

General aspect	Clean and Smooth fur	0
	Some regions with pilo-erection	1
	Pilo-erections	2
	Puffy appearance	3
Posture	Normal	0
	Slightly arched	1
	Arched	2
	Flat	3
Activity	Active	0
	Activity slowed down, but movement still possible	1
	Altered activity, only moves if stimulated	2
	Very impaired activity, does not move after stimulation	3
Response to stimuli	Immediate response to sound and touch	0
	Immediate response to sound, slow response to touch	1
	Slow/no response to sound, sensitive to touch but not moving	2
	No response to sound and touch	3
Eyes	Normal	0
	Eyes not fully open, possible secretions	1
	Eyes closed or not fully opened, secretions	2
	Abundant secretions	3
Total score		

B

Score	0	1	2	3	4
AHAS	< 3	4-5	6-7	8-9	10-11
SBP (mmHg)	> 80	< 80	< 65		
DBP (mmHg)	> 49	< 49			
MAP (mmHg)	> 60	< 60			
HR (BPM)	425-485	400-425 ou > 485	<400		
RR (RPM)	55-65	< 55 ou > 65	< 36 ou > 81		
Leukocytes (10^3/L)	> 4	2-4	< 2		
pH	> 7.2	< 7.2			
Lactates (mmol/L)	< 4	> 4			
Glycemia (mmol/L)	> 6	< 6	< 2		
Creatininemia (µmol/l)	< 20	> 20			
Troponin T (ng/L)	< 25	> 25			
ASAT (UI/L)	< 100	> 100			

To evaluate the impact of endotoxemic shock and treatments on health status, an adapted PRISM score was performed. Rats were monitored frequently throughout the study. The global health status was analyzed by a behavior score (**A**). Physiological functions and circulating parameters were measured (**B**). By summing these data, a PRISM score can be determined all animals. AHAS: Animal Health Assessment Score; SBP: systolic blood pressure; DBP: diastolic blood pressure; MAP: mean arterial pressure; HR: heart rate; RR: respiratory rate; ASAT: aspartate aminotransferase.

Table S2. List of putative cardiac *O*-GlcNAcylated proteins in CTRL, LPS, LPS+R and NButGT groups

Cardiac O-GlcNAcylated proteins were identified by Click-iT®/mass spectrometry approach. CTRL: control group, LPS: i.v. injection of LPS (E. Coli O111:B4, 20 mg/kg), LPS+R: resuscitation with a subcutaneous administration of 10 mL/kg of NaCl solution 0.9% 1h after LPS injection, NButGT: addition of an OGA inhibitor to the resuscitation medium (NButGT: 10 mg/kg). n = 2.

Table S3. List of putative cardiac *O*-GlcNAcylated proteins in CTRL, LPS, LPS+R and NButGT groups with our quality control

Cardiac O-GlcNAcylated proteins were identified by Click-iT®/mass spectrometry approach. Only proteins with at least 15% of coverage, 2 unique peptides and no missing values for each group were retained. CTRL: control group, LPS: i.v. injection of LPS (E. Coli O111:B4, 20 mg/kg), LPS+R: resuscitation with a subcutaneous administration of 10 mL/kg of NaCl solution 0.9% 1h after LPS injection, NButGT: addition of an OGA inhibitor to the resuscitation medium (NButGT: 10 mg/kg). n = 2.

Table S4. Composition of lysis buffer for protein extraction

Chemical product	Final concentration	Volume (µL) for 1mL
**NButGT**	40 µM	2
**PMSF (Sigma)**	1X	10
**Nonidet P40 (Sigma)**		2,5
**Proteases inhibitor complex (Sigma)**	1X	10
**EDTA (pH 8)**	20 µM	40
**PhosSTOP (Roche)**	1X	100
**T.PER (Thermo Scientific)**		QS 1mL

Nonidet 40: 4-Nonylphenyl-polyethylene glycol; PMSF: phenylmethylsulfonyl fluoride; EDTA: Ethylenediaminetetraacetic acid; T.PER: Tissue Protein Extraction Reagent (Bicine 25mM, sodium chloride 150mM (pH 7,6); PhosSTOP: (1R,2S,3R,6S)-1,2-dimethyl-3,6-epoxycyclohexane-1,2-dicarboxylic anhydride, phosphatases inhibitor, NButGT: 1,2-dideoxy-2'-propylalpha-D-glucopyranoso-[2,1-D]-Delta 2'-thiazoline, OGA inhibitor.

Table S5. Antibodies used in western blot analysis

Target	Primary antibody			Secondary antibody	
	Antibody		Dilution	Antibody	Dilution
***O*-GlcNAc**	Ac-RL2-HRP Mouse Monoclonal IgG	201995 Abcam	1/20 000#	/	/
**GFAT1**	Ac-GFAT1 Rabbit Monoclonal IgG	5322 Cell Signaling	1/500*	Anti-rabbit IgG sc 2054 Santa Cruz	1/10 000*
**GFAT2**	Ac-GFPT2 Rabbit Monoclonal IgG	190966 Abcam	1/2000*	Anti-rabbit IgG sc 2054 Santa Cruz	1/10 000*
**OGT**	Ac-OGT Rabbit Polyclonal IgG	24083 Cell Signaling	1/400*	Anti-rabbit IgG sc 2054 Santa Cruz	1/10 000*
**OGA**	Ac-MGEA5 Rabbit Polyclonal IgG	105217 Abcam	1/5000*	Anti-rabbit IgG sc 2054 Santa Cruz	1/10 000*
Tnnc1	Ac-Cardiac Troponin C Rabbit Monoclonal IgG	137130 Abcam	1/2000*	Anti-rabbit IgG sc 2054 Santa Cruz	1/10 000*
Slc25a11	Ac-Slc25a11 Rabbit Polyclonal IgG	155196 Abcam	1/2000*	Anti-rabbit IgG sc 2054 Santa Cruz	1/10 000*
Acly	Ac-ATP citrate lyase Rabbit Monoclonal IgG	40793 Abcam	1/1000*	Anti-rabbit IgG sc 2054 Santa Cruz	1/10 000*
Acly T447 + S451	Ac-ATP citrate lyase T447 + S451 Rabbit Monoclonal IgG	53007 Abcam	1/1000*	Anti-rabbit IgG sc 2054 Santa Cruz	1/10 000*
Acly S455	Ac-ATP citrate lyase S455 Rabbit Polyclonal IgG	4331 Cell Signaling	1/1000#	Anti-rabbit IgG sc 2054 Santa Cruz	1/10 000#

#: dilutions carried out in 3% BSA; *: dilutions carried out in 5% milk.

Table S6. Final result file

Cardiac O-GlcNAcylated proteins were identified by Click-iT®/mass spectrometry approach. CTRL: control group, LPS: i.v. injection of LPS (E. Coli O111:B4, 20 mg/kg), LPS+R: resuscitation with a subcutaneous administration of 10 mL/kg of NaCl solution 0.9% 1h after LPS injection, NButGT: addition of an OGA inhibitor to the resuscitation medium (NButGT: 10 mg/kg). n = 2.
